# Epidemic Enhancement in Partially Immune Populations

**DOI:** 10.1371/journal.pone.0000165

**Published:** 2007-01-17

**Authors:** Juliet R.C. Pulliam, Jonathan G. Dushoff, Simon A. Levin, Andrew P. Dobson

**Affiliations:** 1 Department of Ecology and Evolutionary Biology, Princeton University, Princeton, New Jersey, United States of America; 2 Fogarty International Center, National Institutes of Health, Bethesda, Maryland, United States of America; University of Sheffield, United Kingdom

## Abstract

We observe that a pathogen introduce/pmcdata/journal/plosone/2-2007/1/ingest/pmcmod/sgml/pone.0000165.xmld into a population containing individuals with acquired immunity can result in an epidemic longer in duration and/or larger in size than if the pathogen were introduced into a naive population. We call this phenomenon “epidemic enhancement,” and use simple dynamical models to show that it is a realistic scenario within the parameter ranges of many common infectious diseases. This finding implies that repeated pathogen introduction or intermediate levels of vaccine coverage can lead to pathogen persistence in populations where extinction would otherwise be expected.

## Introduction

The dynamics of pathogen introduction into a naïve, or completely susceptible, host population have been thoroughly described in the literature and are well understood from both theoretical and empirical perspectives [1], [2], [3]. Emerging infectious diseases are generally considered within this framework [4], [5]; however, the possibility of repeated introductions of a “novel” pathogen into a single host population, or of attempts to prophylactically vaccinate populations considered to be at risk for pathogen spillover, requires that we consider the dynamics of infections introduced into pathogen-free populations comprising both susceptible individuals and those with acquired immunity, referred to here as “partially immune” populations. A recent paper by Savill *et al.* used a detailed stochastic model to show that introduction of H5N1 avian influenza into vaccinated poultry populations could promote undetected pathogen persistence, facilitating “silent” spread to neighboring farms [6]. We use very simple dynamical models to demonstrate the generality of this phenomenon.

## Results

### Differential equation model

**Figure 1:**
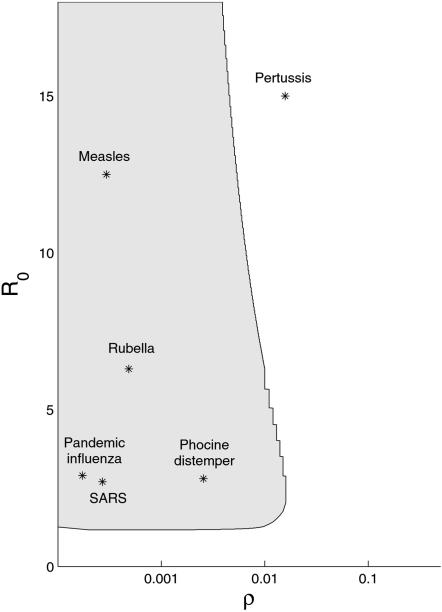
Deterministic prediction of the parameter ranges where epidemic enhancement may be observed. The range of parameter values (grey) for a population size of *N* = 50,000 and the initial condition (*S_0_,I_0_*) = (*N−1, 1*) which demonstrate the behavior of an initial epidemic which dies out (there exists *t>*0 such that *I_t_<*1) followed by persistence upon reintroduction (I*>1), depending on the level of population turnover between pathogen extinction and reintroduction. *R_0_* is the basic reproductive number of the pathogen in a naïve host population; *ρ* is the duration of infectiousness relative to the average duration of immunity. Stars represent parameter values taken from the literature for a variety of common and emerging infectious diseases. Note that the x-axis is shown on a log scale. Parameter values used, estimated ranges of parameter values, and references are given in Table S1.

Damped or sustained oscillations in the number of infectious individuals are a common feature of infectious disease dynamics and are reproduced in even the simplest disease models. For a wide range of realistic parameters, however, differential equation models, which are continuous and deterministic, exhibit a behavior that is generally considered biologically implausible. Although the epidemic caused by pathogen introduction into the naïve host population is self-limiting (that is, after an initial explosion of cases, the susceptible population is depleted to such an extent that the epidemic trajectory dips below the point where a single infectious individual is present in the population), the infection never completely disappears from the population (0*<I<<*1) due to the treatment of individuals as continuous in this type of model. Instead, the susceptible population is replenished following the initial epidemic and eventually reaches a level where the effective reproductive number of the pathogen in the population, *R_e_*, is greater than one. A new epidemic occurs, which is longer in duration than the initial epidemic but has a lower epidemic peak. In this manner, the system eventually reaches an endemic equilibrium where the pathogen persists (I*>1). The key parameters which determine whether an infection will exhibit this behavior are the host population size (*N*), the basic reproductive number of the pathogen in the host population (*R_0_*), and the ratio (*ρ*) of the duration of infectiousness to the average duration of immunity. Figure 1 shows the region of (*ρ,R_0_*) parameter space over which the number of infectives falls below one after an initial epidemic for a population of 50,000 individuals.

### Stochastic model

**Figure 2:**
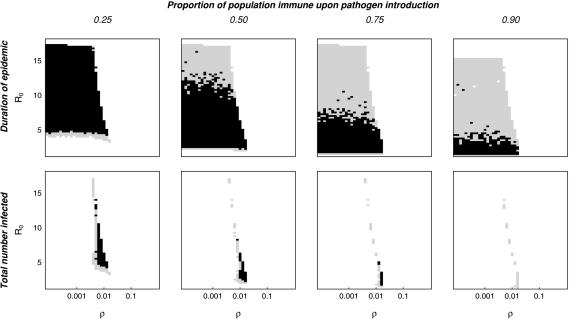
Stochastic demonstration of epidemic enhancement in partially immune populations. Each plot is a comparison of the outcome of the runs starting with a particular immunity level to the 90^th^ percentile of runs in a naïve population. Black spaces show where enhancement occurs; that is, >15% of epidemics in partially immune population are longer (top row) or larger (bottom row) than the 90^th^ percentile of epidemics in a naïve population. Grey spaces show the area where 5–15% of the epidemics in the partially immune population are longer or larger than the 90^th^ percentile of epidemics in a naive population (so vaccination has little or no effect in either direction). The simulation was run 500 times for introduction into a naïve population for each set of parameter values, and these runs were used to determine the 90^th^ percentile for epidemic duration and size; the pathogen was considered to persist if it remained in the population for ≥300 disease generations. The simulation was then run 500 times for each of the levels of initial population immunity (25%, 50%, 75%, 90%) in the portion of parameter space for which the pathogen did not persist in the naïve population. The total population size is *N* = 50,000.

**Figure 3:**
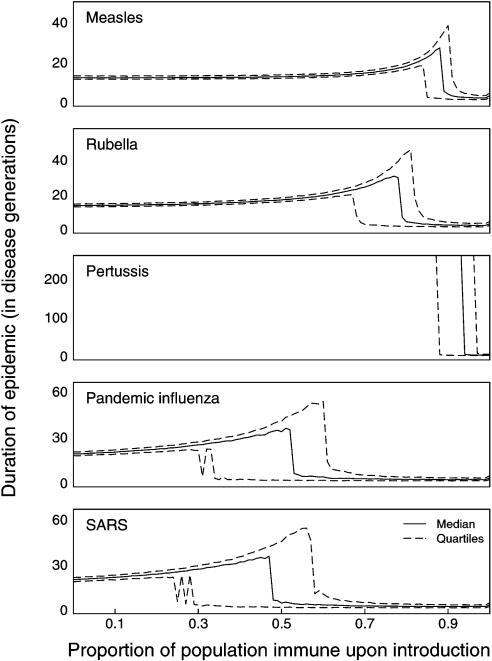
Enhancement of epidemic duration for diseases in human populations. Epidemic duration in a population of *N* = 50,000 individuals for a variety of human pathogens as a function of population immunity at introduction. Solid lines show the median duration in disease generations for 1,000 simulation runs at each level of initial population immunity; dashed lines show quartiles. Each pathogen shows some level of enhancement of epidemic duration with increased immunity except pertussis. Enhancement of epidemic size is not observed for these pathogens for *N* = 50,000.

Although results of the differential equation model are dependent on the treatment of individuals as continuous entities, analogous behavior is observed in stochastic systems with discrete individuals when a pathogen is introduced or reintroduced into a partially immune population. The presence of immune individuals in the population results in a lower force of infection upon introduction or reintroduction of the pathogen. Instead of burning through the susceptible population and driving itself to extinction as in a naïve population, the pathogen produces a smaller epidemic with a less dramatic depletion of individuals, which may in turn allow the pathogen to persist. Within the region of parameter space where this occurs, the level of population immunity present at the time of introduction will determine whether spillover leads to no epidemic, a self-limiting epidemic, or long-term persistence. Introduction into a partially immune population with intermediate levels of population immunity will often lead to an epidemic of longer duration and/or higher total number of infectious individuals than introduction into a naïve population, a phenomenon which we call “epidemic enhancement.” Figure 1 illustrates that a variety of common and emerging infectious diseases fall within the region of parameter space where enhancement is expected. This suggests that multiple introductions may have been required for some or all of these diseases to establish in the human population. Stochastic model results (Figures 2 & 3) suggest that enhancement of epidemic duration is common throughout this region of parameter space, whereas enhancement of epidemic size is only observed where equilibrium populations of infectious individuals are sufficient to allow long-term persistence without stochastic extinction.

## Discussion

Epidemic enhancement may have important implications for emerging infectious diseases, particularly because pathogen persistence within an isolated population will increase the time span over which movement of individuals may spread the disease to new areas and may also decrease the probability that infection will be detected or diagnosed [6]. Of primary concern is the possibility that prophylactic vaccination would promote pathogen persistence in populations with high turnover rates, such as on domestic livestock and poultry farms, because large-scale vaccination efforts are unlikely to vaccinate frequently enough to maintain herd immunity within farm populations characterized by rapid turnover. As discussed by Savill *et al.*, incomplete vaccine efficacy would lead to similar dynamics [6].

Finally, we must be aware that the same effect can be produced by repeated introductions of a pathogen into a single population. Thus, populations which are known to have been infected with a pathogen of interest must be considered to pose substantial risk even if they are currently pathogen-free. In 1998, the reintroduction of Nipah virus onto a previously exposed pig farm appears to have been responsible for the long-term persistence of the virus, which eventually led to widespread infection in pigs and people (J. Pulliam, unpublished results). Although this is the only example known to us in which enhancement has facilitated the spread of an infectious agent into human populations, the mathematically robust nature of this phenomenon suggests that we must take the possibility of enhancement into account when considering the prevention and control of emerging diseases.

## Materials and Methods

### Pathogen dynamics in a simple differential equation *SIR* model

#### Model structure

The following model is used to explore the dynamics of pathogen introduction into partially immune populations:

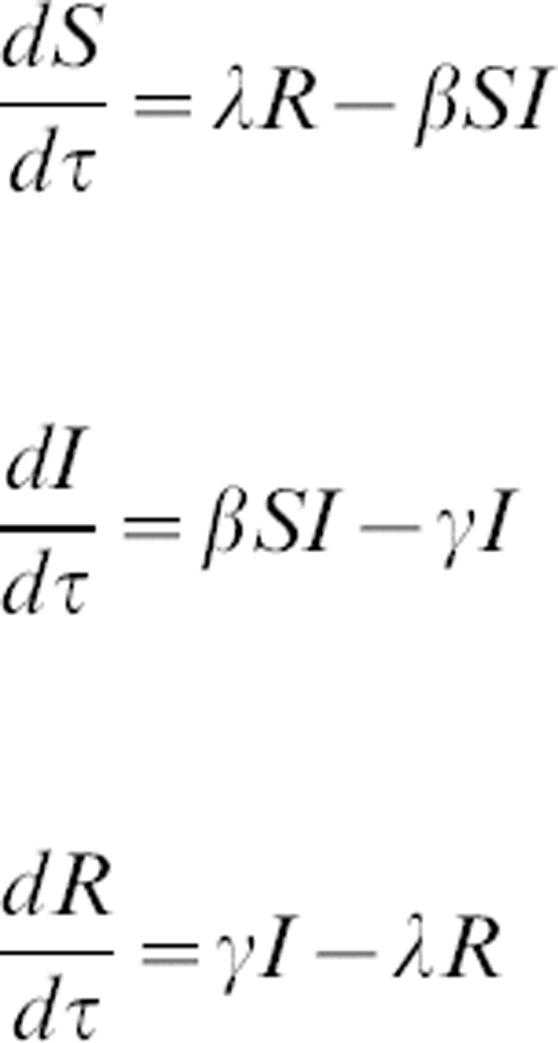

where *S* is the number of susceptible individuals, *I* is the number of infectious individuals, *R* is the number of recovered (immune) individuals, *γ* is recovery rate (*γ* = D^−1^, where *D* is the average duration of infection), *λ* is turnover rate of the immune population, and *β* is the transmission coefficient. The total population size, *N* = *S*+*I*+*R*, remains constant, so only two equations are needed to describe the entire system. Rescaling time to be in units of the infectious period (i.e.,*t = γτ*), and recognizing that the basic reproductive ratio (the number of infectious individuals produced by a single infectious individual in a naïve population) is 
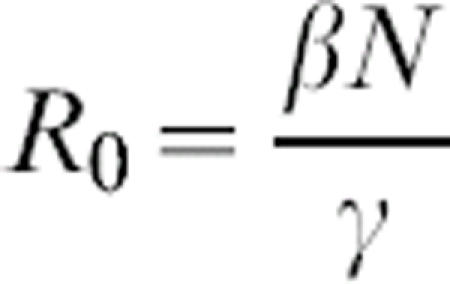
 , gives:
1

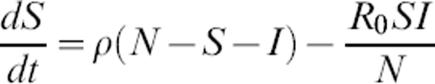


2

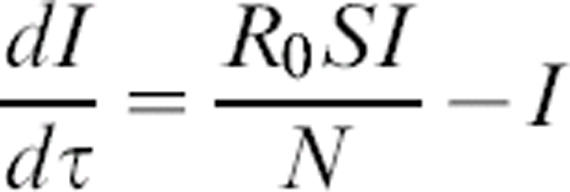

where 
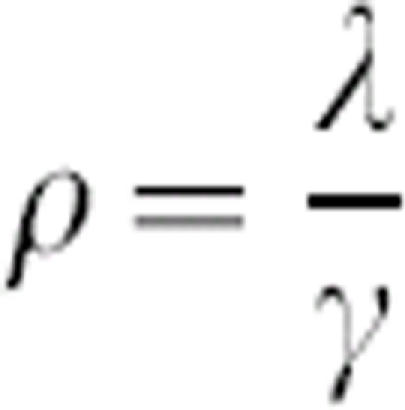
 is the rescaled turnover rate. The system has two equilibria: a trivial disease-free equilibrium (S* = N, I* = 0) and an endemic equilibrium at

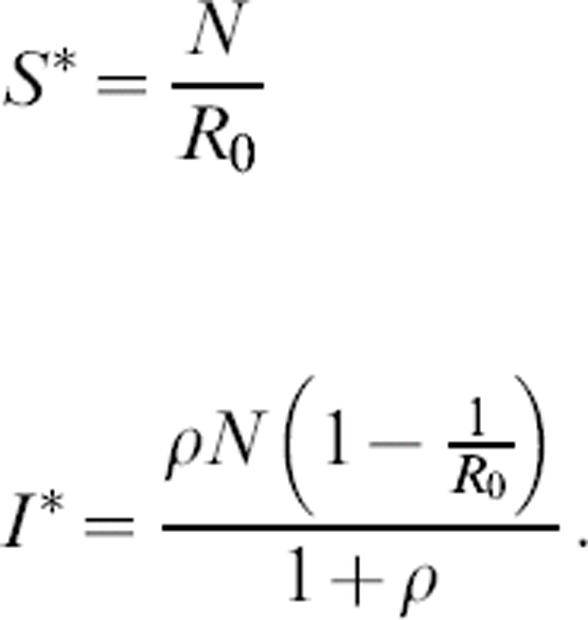




#### Model assumptions

Several key assumptions should be noted:

1. The population is well-mixed, and all individuals in the population are identical, except with regard to infection status (e.g., there is no age structure).

2. Transmission is density dependent.

3. Waiting times for all events are exponentially-distributed.

4. Both time and the number of individuals in each class are continuous variables.

5. There is no mortality due to infection.

6. There is complete overlap between “infected” and “infectious” individuals (i.e., there is no incubation period before infectivity and there are no latent infections).

7. The duration of infection is sufficiently short that background mortality in the infectious class can be ignored. Death is explicit only in the recovered class and dying individuals are immediately replaced by birth of susceptible individuals, keeping population size constant (this could also be viewed as an immigration/emigration process or as loss of immunity in a constant population). This assumption should be modified when considering diseases with particularly long infectious periods, such as many STD's.

#### Stability of equilibria

The endemic equilibrium is stable whenever *R*_0_>1, with damped oscillations observed in the region where *R*_0_>1 and

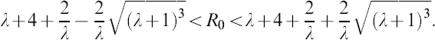




When *R*_0_<1, the endemic equilibrium is a saddle point. The disease-free equilibrium is a stable node when *R*_0_<1 and a saddle point when *R*_0_>1.

### Stochastic implementation of the *SIR* model

Although the ODE model is a useful tool for understanding the dynamics of pathogen introduction, the treatment of individuals as continuous entities can produce strange results. This assumption can easily be discarded by developing a stochastic analogue to the ODE model with discrete individuals, after [7] (the other assumptions described above remain).

The stochastic model takes the following form, in which time is rescaled as above (see Equations 1 & 2):

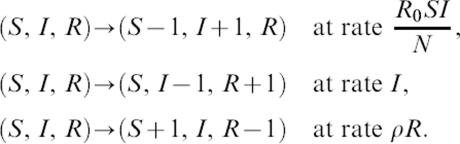




### Disease parameters

The parameters *R_0_* and *ρ* were derived from estimates in the literature. The rescaled turnover rate, 
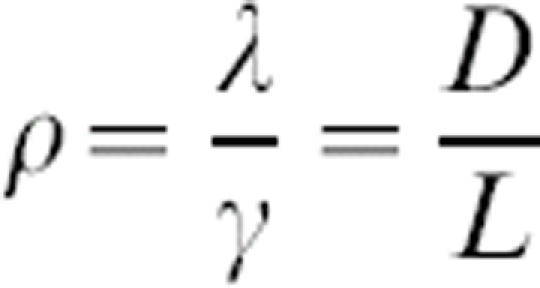
 , was calculated as the ratio of *D* (the expected duration of the infectious period) to *L* (the average duration of immunity), where the parameter values given were first converted to days. For human diseases producing lifelong immunity, *L* was assumed to be 65 years. Table S1 gives the parameter values used and lists the appropriate references.

## Supporting Information

Table S1 Parameter values and references. Table S1 gives parameter values used, estimated ranges of parameter values, and appropriate references.
(0.05 MB DOC)

## References

[1] Kermack WO, McKendrick AG (1927). A contribution to the mathematical theory of epidemics.. Proc R Soc London Ser A.

[2] Anderson RM, May RM (1986). The invasion, persistence and spread of infectious diseases within animal and plant communities.. Philos Trans R Soc London Ser B.

[3] Diekmann O, Heesterbeek JAP, Metz JAJ (1990). On the definition and the computation of the basic reproduction ratio *R*0 in models for infectious diseases in heterogeneous populations.. J Math Biol.

[4] Dobson A, Foufopoulos J (2001). Emerging infectious pathogens of wildlife.. Philos Trans R Soc London Ser B.

[5] May RM, Gupta S, McLean AR (2001). Infectious disease dynamics: what characterizes a successful invader?. Philos Trans R Soc London Ser B.

[6] Savill NJ, St-Rose SG, Keeling MJ, Woolhouse MEJ (2006). Silent spread of H5N1 in vaccinated poultry.. Nature.

[7] Gillespie DT (1977). Exact stochastic simulation of coupled chemical reactions.. J Phys Chem.

