# Supplementary material for: Epidemic Enhancement in Partially Immune Populations
Source: PLoS One. 2007 Jan 17;2(1):e165. doi: 10.1371/journal.pone.0000165 (PMC1769520; doi:10.1371/journal.pone.0000165)
Supplement: Table S1 — Parameter values and references. Table S1 gives parameter values used, estimated ranges of parameter values, and appropriate references. (0.05 MB DOC) [file pone.0000165.s001.doc]

| *Pathogen* | *Parameter* | *Estimated range* | *Value used* | *References* |
| --- | --- | --- | --- | --- |
| **Measles** | *R_0_* | 12.5, 13.7-18.0 | 12.5 | [1] |
|  | $D=1/\gamma$ | 6-7 days | 7.0 days | [1] |
|  | $L=1/\lambda$ | -- | 65 years | -- |
| **Rubella** | *R_0_* | 6.0, 6.7 | 6.3 | [1] |
|  | $D$ | 11-12 days | 11.5 days | [1] |
|  | $L$ | -- | 65 years | -- |
| **Pertussis** | *R_0_* | 12.2, 14.3-17.1 | 15.0 | [1] |
|  | $D$ | 21-23 days | 23 days | [1] |
|  | $L$ | 4-20[[1]](#footnote-2) years | 4 years | [2] |
| **Pandemic influenza (A/H1N1)** | *R_0_* | 2.9-3.9 | 2.9 | [3] |
|  | $D$ | -- | 4.1 days | [3] |
|  | $L$ | -- | 65 years | -- |
| **SARS** | *R_0_* | ≈ 2.7-3 | 2.7 | [4,5] |
|  | $D$ | (95% CI: 5.2-7.7) | 6.4 | [6] |
|  | $L$ | -- | 65 years | -- |
| **Phocine distemper** | *R_0_* | -- | 2.8 | [7] |
|  | $D$ | -- | 7 days | [7] |
|  | $L$ | 5-10 years | 7.5 years | [7] |

**References**

1. Anderson RM, May RM (1982) Directly transmitted infectious diseases: control by vaccination. Science 215:1053-1060.

2. Wendelboe AM, Van Rie A, Salmaso S, Englund JA (2005) Duration of immunity against pertussis after natural infection or vaccination. Pediatr Infect Dis J 24(Supplement)S58-S61.

3. Mills CE, Robins JM, Lipsitch M (2004) Transmissibility of 1918 pandemic influenza. Nature 432:904-906.

4. Lipsitch M, Cohen T, Cooper B, Robins JM, Ma S, et al. (2003) Transmission dynamics and control of severe acute respiratory syndrome. Science 300:1966-1970.

5. Riley S, Fraser C, Donnelly CA, Ghani AC, Abu-Raddad LJ, et al. (2003) Transmission dynamics of the etiological agent of SARS in Hong Kong: impact of public health interventions. Science 300:1961-1665.

6. Donnelly CA, Ghani AC, Leung GM, Hedley AJ, Fraser C, et al. (2003) Epidemiological determinants of spread of causal agent of severe acute respiratory syndrome in Hong Kong. Lancet 361:1761-1766.

7. Swinton J, Harwood J, Grenfell BT, Gilligan CA (1998) Persistence thresholds for phocine distemper virus infection in harbour seal *Phoca vitulina* metapopulations. J Anim Ecol 67:54-68.

1. This range is for naturally-acquired immunity produced by active infection; protective immunity from vaccination wanes after 4 -12 years. The extreme value of 4 years was chosen for simulations to provide maximum contrast to the pathogens that produce lifelong immunity. [↑](#footnote-ref-2)
